# *Bacillus subtilis* MBI600 Promotes Growth of Tomato Plants and Induces Systemic Resistance Contributing to the Control of Soilborne Pathogens

**DOI:** 10.3390/plants10061113

**Published:** 2021-05-31

**Authors:** Anastasios Samaras, Efstathios Roumeliotis, Panagiota Ntasiou, George Karaoglanidis

**Affiliations:** 1 Plant Pathology Laboratory, Forestry and Natural Environment, Faculty of Agriculture, Aristotle University of Thessaloniki, P.O. Box 269, 54124 Thessaloniki, Greece; samarasanast@gmail.com (A.S.); ntasioup@agro.auth.gr (P.N.); 2 Department of Agriculture, Theodoropoulou Terma, University of Patras, 27200 Amaliada, Greece; stathisroumel@gmail.com

**Keywords:** auxin-related genes, *Fusarium oxysporum* f.sp. *radicis-lycopersici*, Induced Systemic Resistance, JA/ET signaling, *Pythium ultimum*, *Rhizoctonia solani*, SA signaling

## Abstract

*Bacillus subtilis* MBI600 (*Bs* MBI600) is a recently commercialized plant-growth-promoting rhizobacterium (PGPR). In this study, we investigated the effects of *Bs* MBI600 on the growth of tomato and its biocontrol efficacy against three main soilborne tomato pathogens (*Rhizoctonia solani*, *Pythium ultimum*, and *Fusarium oxysporum* f.sp. *radicis-lycopersici-Forl*). Furthermore, the root colonization ability of the *Bs* MBI600 strain on tomato roots was analyzed in vivo with a yellow fluorescence protein (*yfp*)-labeled strain, revealing strong colonization ability, which was affected by the root growth substrate. The application of *Bs* MBI600 on tomato plants resulted in significant increases in shoot and root lengths. Transcriptional activation of two auxin-related genes (*SiPin6* and *SiLax4*) was observed. Single applications of *Bs* MBI600 on inoculated tomato plants with pathogens revealed satisfactory control efficacy compared to chemical treatment. Transcriptomic analysis of defense-related genes used as markers of the salicylic acid (SA) signaling pathway (*PR-1A* and *GLUA*) or jasmonic acid/ethylene (JA/ET) signaling pathway (*CHI3*, *LOXD*, and *PAL*) showed increased transcription patterns in tomato plants treated with *Bs* MBI600 or *Forl*. These results indicate the biochemical and molecular mechanisms that are activated after the application of *Bs* MBI600 on tomato plants and suggest that induction of systemic resistance (ISR) occurred.

## 1. Introduction

The tomato plant (*Lycopersicum esculentum Mill.*) belongs to the *Solanaceae* family and is the most important commercial vegetable crop cultivated worldwide, either for fresh consumption or industrial processing. Based on FAO statistics, the world production has been consistently increasing during the last two decades, which reached a value of around 180 million tons of fruit for either fresh consumption or processing during 2020 [1]. The sustainability of tomato production is hampered by several diseases caused by fungal, oomycete, bacterial, or viral pathogens. Soilborne diseases caused by fungal and oomycete pathogens are highly destructive under conditions favorable for their development. Among these soilborne diseases, Fusarium crown and root rot (FCRR) caused by *Fusarium oxysporum* f.sp. *radicis-lycopersici* Schlecht. (*Forl*), and Rhizoctonia or Pythium damping-off caused by *Rhizoctonia solani* Kühn (teleomorph: *Thanatephorus cucumeris* (Frank) Donk) and *Pythium* spp., respectively, are the most destructive, having a worldwide distribution. FCRR caused by *Forl* occurs in field, greenhouse, and hydroponic cultures [2]. *Forl* does not have a known sexual stage, while it infects the main root and the crown of the plants, causing root or crown rots and vascular necrosis that lead to wilting and subsequently to the death of the plants [3]. *R. solani* is a *Basidiomycete* fungus that lives in the soil, forming microsclerotia but not asexual spores. It has an extremely wide host range and on most of its hosts causes damping-off and root rot of seedling plants [4]. Similarly, *P. ultimum* Trow is a damping-off pathogen infecting the root systems of tomato plants, causing an initial weakening of the plants and subsequent root rot, which may lead to plant death [5].

Currently, the control of these soilborne pathogens is based on cultural methods such as crop rotation or soil solarization, use of resistant varieties (if available), and chemical control [6,7,8]. The use of resistant varieties could be the most economic and long-term approach to combat these diseases. However, although significant advances have been made related to the development of tomato varieties with resistance to *Forl*, the availability of varieties resistant to soilborne pathogens such as *R. solani* or *Pythium* spp. is null [2]. Chemical control of these soilborne pathogens has shown several limitations, such as the reduced number of effective fungicide products, their low efficacy, and issues related to social concerns for pesticide residues or environmental pollution. Thus, the available cultural or chemical control methods or the use of resistant varieties do not ensure sustainable tomato production, and hence the development of alternative control measures is a necessity.

During recent decades, the biological control of soilborne tomato pathogens has attracted research interest. Numerous fungal and bacterial antagonist species have been tested against these pathogens and several of them have already been commercialized [5,8,9,10]. Among these, rhizosphere-associated, plant-growth-promoting rhizobacteria (PGPR) have been explored as biocontrol agents (BCAs) during the last 40 years and they represent a rapidly expanding branch of the crop protection industry—biopesticide products [11]. PGPR strains can be effective against plant pathogens by exploiting several mechanisms of action, such as antagonism, production of antibiotics, competition for nutrients or space, and the induction of systemic resistance (ISR) [12]. Early attempts to unravel ISR induction by PGPR suggested that it was mediated through the enhancement of ethylene and jasmonic acid (ET/JA) signaling pathways, which confer resistance to necrotrophic pathogens [13,14]. However, recent evidence was provided suggesting that enhancement of the salicylic acid (SA) signaling pathway associated with plant resistance to biotrophic pathogens may mediate ISR in PGPR-treated plants [13,15].

An additional major characteristic of PGPR, other than their contribution in combating plant pathogens, is their influence on plant growth [16]. This influence is mediated through a combination of several mechanisms, including phytostimulation with direct production of phytohormones by the PGPR and supply to the plant, indirect contribution by stimulation of phytohormone production by the plants, alterations in the root-system architecture, increases in nutrient availability, and increases in root permeability [16,17,18,19].

*Bacillus* strains belonging to several species within the genus are by far the most important PGPR group that have been tested as potential biocontrol agents, which were registered for use on several crops. Their extensive use is based on their outstanding characteristics, such as their increased tolerance to stress conditions, their endospore formation ability, the high root colonization ability they exhibit, and the vast number of secondary metabolites they produce, conferring high biocontrol potential [20,21]. In the recent past, several *Bacillus* spp. strains have been evaluated for their effects on the control of soilborne tomato diseases, such as Rhizoctonia or Pythium damping-off, Fusarium wilt, or FCRR [9,22,23,24].

*B. subtilis* MBI600 (thereafter *Bs* MBI600) is a BCA that was commercialized recently by BASF throughout the world. In a study by our group, its taxonomy was unraveled through whole-genome sequence, and several genes associated with plant growth promotion, root colonization ability, and biological control of plant pathogens were identified [25]. Although it is already registered for use in several crops against a wide array of fungal and bacterial pathogens, detailed information on its effects against specific pathogens is limited. On rice it has been found to be effective against *Rhizoctonia solani* [26], while recently our group showed that it was effective against two major soilborne pathogens of cucumber, *Fusarium oxysporum* f.sp. *radicis-cucumerinum*, the agent of Fusarium crown and root rot of cucumber, and *Pythium aphanidermatum,* the agent of Pythium damping-off of cucumber [25]. For tomato plants, data on the efficacy of *Bs* MBI600 against fungal and oomycete pathogens is limited, however high efficacy has been reported against two major viral diseases, TSWV and PVY [27]. Previous research performed in our laboratory for the requirements of product registration showed that double application of *Bs* MBI600 as a soil drench just after seed sowing and 10 days later can ensure high efficacy against FCRR caused by *Forl* [28]. 

Despite the fact that *Bs* MBI600 has recently been commercialized, information on its biocontrol activity on tomato plants and the mechanisms associated with this is restricted mostly to viral pathogens [15,27]. Similarly, there is no available information on the root colonization ability of tomato plants grown on different substrates or related to the mechanisms of growth induction on tomato plants. Such information is crucial for the optimization of BCA use in agricultural practice. Therefore, the current study was initiated to: (a) investigate the biocontrol ability of *Bs* MBI600, in vitro and in planta, against 3 soilborne fungal and oomycete tomato pathogens (*F. oxysporum* f.sp. *radicis-lycopersici* (*Forl*), *R. solani* and *P. ultimum*); (b) determine its ability to colonize tomato roots grown in different growth substrates by taking advantage of the chloramphenicol-resistant cassette inserted in the *yfp*-plasmid; (c) investigate the expression of defense- and auxin-related genes in tomato plants, after treatment with *Bs* MBI600 in the presence and the absence of *Forl*, as a typical soilborne tomato pathogen.

## 2. Results

### 2.1. In Vitro Antagonistic Activity of Bs MBI600 against Forl, P. ultimum, and R. solani

Antagonistic activity of *Bs* MBI600 was tested on PDA dual cultures, a nutrient medium suitable for the growth of all the microorganisms used in the study. After seven days of dual culturing with the three different plant pathogens, *Bs* MBI600 reduced the mycelial growth of them at variable rates (Table 1). The relative inhibition of mycelial growth for *Forl*, *P. ultimum* and *R.solani* in the presence of *Bs* MBI600 was 64.1, 27.8 and 7.2%, respectively. In addition, a strong (++) inhibition zone of mycelial growth between the fungal and bacterial colonies was observed in the dual cultures with *Forl* (++), a less intense inhibition zone was observed in the dual cultures with *R. solani* (+), while there was not inhibition zone in the dual cultures with *P. ultimum* (Table 1).

### 2.2. Growth Characteristics of Tomato Plants Treated with Bs MBI600

**Figure 1:**
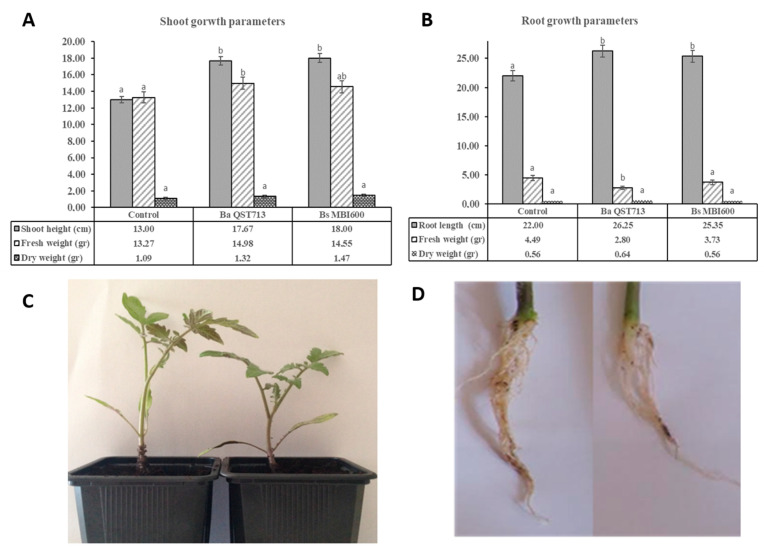
Effects of *Bacillus subtilis* MBI600 applications on tomato plant (cv. Belladonna) shoot (**A**) and root (**B**) growth parameters compared to the growth of untreated control plants and *Bacillus amyloliquefaciens* QST713-treated plants (reference biological treatment). Different letters in the columns indicate significant differences between the treatments according to Fisher’s LSD test (*p* < 0.05). Vertical lines indicate the standard error of the mean. (**C**) *Bs* MB 600-treated (left side) and untreated plants (right side), showing differences in shoot lengths. (**D**) *Bs* MBI600-treated (left side) and untreated plants (right side), showing differences in root lengths.

To determine the effect of *Bs* MBI600 treatments on the growth promotion of tomato plants, pot experiments were conducted. Measurements of the growth parameters on tomato plants, 35 days after sowing and incubation under greenhouse conditions, showed that application of both *Bs* MBI600 and *Ba* QST713 (*Bacillus amyloliquefaciens* QST713) resulted in significant (*p* < 0.05) increases in shoot height and root length compared to the untreated control plants (Figure 1). The mean shoot and root lengths of *Bs* MBI600-treated plants were measured as 18 and 25.35 cm, respectively, while the respective values for untreated plants were 13 and 22 cm (Figure 1). In contrast, no significant differences (*p* > 0.05) were observed between the control and *Bs* MBI600-treated plants regarding fresh and dry weights of shoot and root samples (Figure 1).

### 2.3. Root Colonization

The counts of bacterial cells on the chloramphenicol-amended medium showed that the *Bs* MBI600 strain was able to successfully colonize tomato roots in all 4 different growth substrates tested, although with ranging effectiveness between these different systems. In all 4 substrates, the higher cfu numbers for *Bs* MBI600 were measured at five days post-application (dpa), while declines in the cfu numbers in the following sampling dates were observed (Table 2). At this first sampling time, for all treatments the population density counts were found to be lower compared to the initial population rate of 2 × 10^10^ cfu cm^−1^. At 5 dpa, a higher count for bacterial cells was measured in the commercial peat mixture, with a value of 3.2 × 10^5^ cfu cm^−1^, followed by 3 × 10^5^ cfu cm^−1^ for the hydroponic cubes and 2 × 10^5^ cfu cm^−1^ for the gnotobiotic system (Table 2). However, assessments conducted 15 dpa showed that the colonization pattern changed and a rapid decline in the cfu numbers for *Bs* MBI600 was observed for the commercial peat mixture substrate, with a rate of 4 × 10^2^ cfu cm^−1^ (Table 2). A further decline in the *Bs* MBI600 population on tomato roots was observed at 20 dpa. At this sampling point, population rates ranged from 1.7 to 4 × 10^2^ cfu cm^−1^, without significant differences (*p* > 0.05) among treatments (Table 2). 

### 2.4. Biocontrol Activity of Bs MBI600 against Soilborne Tomato Pathogens

**Figure 2:**
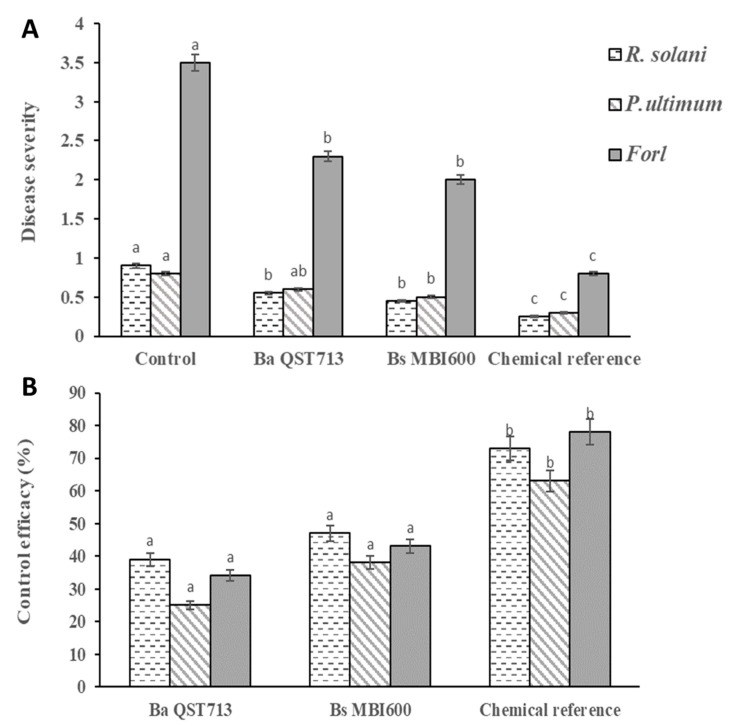
Disease severity (**A**) and control efficacy (%) (**B**) in tomato plants (cv. Belladonna) artificially inoculated with *Rhizoctonia solani, Pythium ultimum,* or *Fusarium oxysporum* f.sp. *radicis-lycopersici* and treated with the biological control agent *Bacillus subtilis* MBI600 (*Bs* MBI600). *Bacillus amyloliquefaciens* QST713 (*Ba* QST713) and 8-hydroxyquinoline or hymexazol were the commercial biological and chemical reference treatments, respectively. Disease severity values for *Forl* and for *R. solani* or *P.ultimum* were measured based on 0–4 and 0–1 disease index scales, respectively. Mean values followed by different letters in the column are significantly different between the applications at *p* = 0.05, according to Fisher’s LSD test. Bars in the columns indicate the standard error of the mean obtained from two independent replications.

In planta measurements of the efficiency of *Bs* MBI600 in controlling the three soilborne tomato pathogens after a single application showed that it possesses the ability to reduce disease severity by 40–50%. The application of *Bs* MBI600 resulted in a significant reduction (*p* < 0.05) of disease severity compared to that observed in the untreated control treatment, caused by all three pathogens tested (Figure 2A). Lower (*p* < 0.05) disease severity values were observed on plants treated with the chemical reference products. Disease severity values for all the three pathogens tested on plants treated with the biological reference treatment were similar (*p* > 0.05) to those observed on *Bs* MBI600-treated plants (Figure 2A). The control efficacy values achieved by the *Bs* MBI 600 application ranged from 38 to 47% and were similar to those achieved by the standard biological reference treatment of *Ba* QST713 (Figure 2B). Higher control efficacy values, which ranged from 63 to 78%, were achieved with the two standard chemical treatments (Figure 2B).

### 2.5. Induction of Auxin-Related Genes in Tomato Plants Treated with Bs MBI600

**Figure 3:**
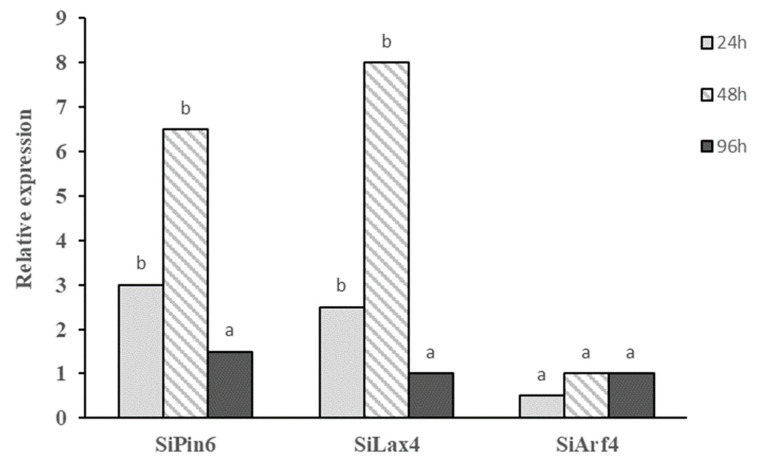
Transcript levels of auxin-related genes (*SiPin6*, *Silax4*, and *SiArf4*) in tomato plants (cv. Belladonna) after treatment with *Bacillus subtilis* MBI 600. Expression levels were analyzed by qRT-PCR at 24, 48, and 96 h post-application compared to untreated plants. The cDNA samples were normalized using the endogenous *cox* gene. Different letters on the columns indicate significant differences at the three time points according to Tukey’s test at *p* = 0.05.

Besides the impact of *Bs* MBI600 on plant growth characteristics, and in particular on shoot and root lengths, we investigated the expression levels of three auxin-related genes of tomato plants. *Bs* MBI600 treatment promoted the expression of the auxin-related genes in tomato compared to the untreated control plants. Among the three measured genes, the expression of *SiArf4* was not found to have changed significantly at any of the three sampling times (Figure 3). In contrast, for the remaining two auxin-related genes, induction of transcript levels was activated 24 hours post-application. At this time point, 3- and 2.8-fold changes were observed in the relative expression levels of *SiPin6* and *SiLax4*, respectively. The highest expression patterns for both genes were measured at 48 h post-application, with 6.5- and 8-fold changes, respectively (Figure 3). However, for both genes, their relative expression rates declined significantly (*p* < 0.05) at 96 h post-application of *Bs* MBI600 (Figure 3). 

### 2.6. Induction of Defense-Related Genes in Tomato Plants Treated with Bs MBI600

**Figure 4:**
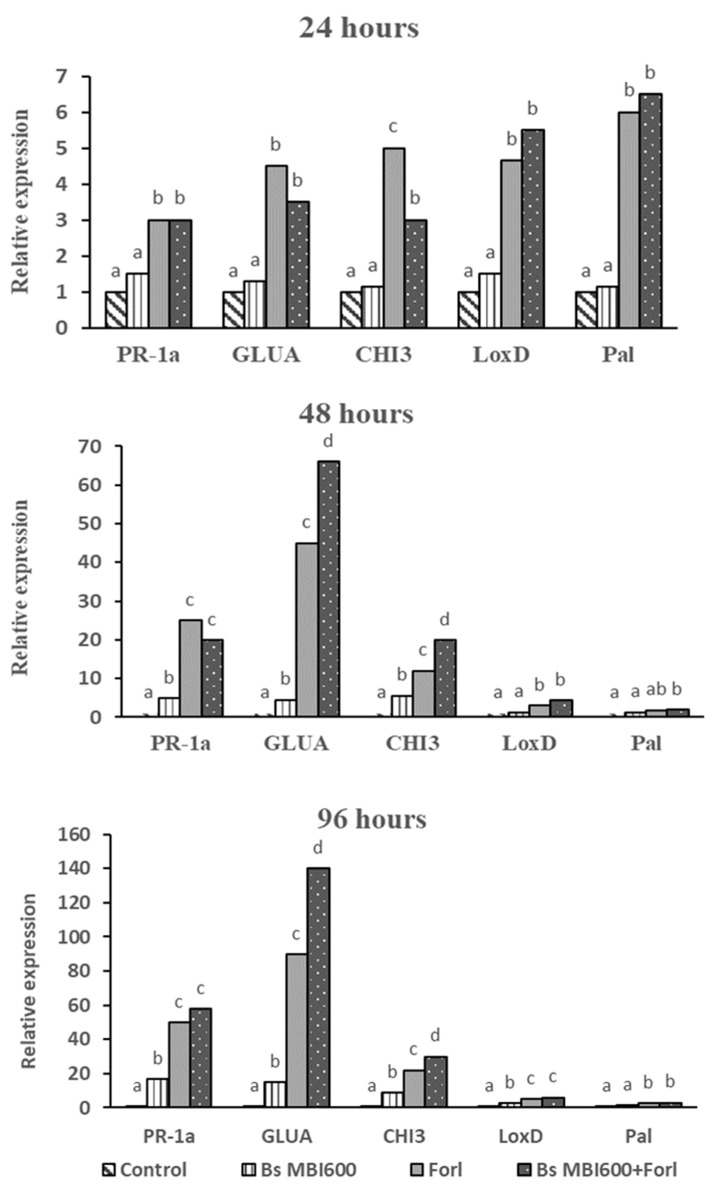
Expression levels of defense-related genes (*PR-1a*, *GLUA*, *CHI3*, *LoxD* and *Pal*) in tomato plants (cv. Belladonna) after treatment with *Bacillus subtilis* MBI 600 (Bs MBI600), inoculation with *Fusarium oxysporum* f.sp. *radicis-lycopersici* (Forl), combined application of both microorganisms (*Bs* MBI600-Forl) and untreated or non-inoculated plants (control). Expression levels were evaluated by qRT-PCR at 24, 48, and 96 h post-inoculation and the cDNA samples were normalized using the endogenous *cox* gene. Different letters in the columns indicate significant differences between the different treatments according to Tukey’s test (*p* < 0.05).

To determine whether the moderate efficacy against *Forl* observed by *Bs* MBI600 treatment was associated with the induction of defense-related genes, five genes were selected (*PR-1a*, *GLUA*, *CHI3*, *LoxD*, and *Pal*) and their expression levels were analyzed using qRT-PCR. The relative expression levels of the five defense-related genes at 24, 48, and 96 h post-application are summarized in Figure 4. Overall, the application of *Bs* MBI600 did not induce increased expression of any defense-related gene at 24 h post-application. In contrast, at the same time point, artificial inoculation with *Forl* resulted in increased (*p* < 0.05) induction rates for all the five genes tested, ranging from a 3-fold increase for *PR-1a* to 6-fold for *Pal* (Figure 4). Similarly, the combined application of *Bs* MBI600 and artificial inoculation with *Forl* resulted in an increase of the gene expression at levels similar to those observed when only *Forl* was applied to the tomato plants. Measurements of gene expression levels 48 h after the application of *Bs* MBI600 showed that it caused 5-, 4.5-, and 5.6-fold increases of *PR-1a*, *GLUA*, and *CHI3* expression, respectively, while *LoxD* and *Pal* expression remained unchanged in plants that received only *Bs* MBI600 treatment. At the same time point, in plants that had been inoculated with *Forl*, the transcript levels still showed an upward trend over time. The highest induction rates were observed for *PR-1a*, *GLUA*, and *CHI3* with 25-, 45-, 12-fold increases, respectively, while for *GLUA* and *CHI3* the induction rates were even higher in plants that received both *Bs* MBI600 application and artificial inoculation with *Forl*. Interestingly, in plants that received only the *Bs* MBI600 application, the highest transcript levels for *PR-1a*, *GLUA*, *CHI3*, *LoxD*, and *Pal* with 17-, 15-, 9-, 2.5- and 1.6-fold changes, respectively, were observed at 96 hours post-application. At the same time point of 96 h post-application, the highest transcript levels for all five genes were observed in plants inoculated with *Forl* and plants that received the combined application of *Bs* MBI600 and artificial inoculation with the pathogen. Interestingly, the expression levels for *GLUA* and *CHI3* genes in plants that received the combined application of *Bs* MBI600 and *Forl* were significantly higher (*p* < 0.05) compared to the respective expression levels in plants that received only the artificial inoculation with the pathogen. For instance, the GLUA transcript levels in plants that had been treated only with *Forl* showed a 90-fold increase compared to the control plants, while a 140-fold increase was observed in the GLUA transcript levels of plants that received the combined application of *Forl* and *Bs* MBI600. 

## 3. Discussion

Biological control of plant diseases has been established as a promising tool to overcome the limitations of other disease control methods or even the absence of other control methods. In this study, the effects of a recently commercialized PGPR strain on the growth of tomato plants and its ability to control certain major fungal and oomycete soilborne pathogens of this crop were evaluated. Our data showed that *Bs* MBI600 could be an effective BCA for use in tomato crops, both for the induction of plant growth and for the control of major soilborne pathogens. In our experimental design, *Bs* MBI600 was applied only once via soil drenching, which provided moderate control efficacy against all three pathogens tested. In a previous study conducted by our group for registration purposes, *Bs* MBI600 showed higher efficacy against *Forl*. However, in that experimental design, *Bs* MBI600 was used in a dual application scheme [28]. Previous studies have shown that the efficacy of biopesticides can be increased with an increased number of applications, shorter spray intervals, or the combined use of different biopesticides [9,29]. Furthermore, in addition to the number of applications, the application dose may play a crucial role in the efficacy of BCAs, as was recently shown for *Bs* MBI600 against viral diseases in tomato plants [15]. Therefore, further research is required to optimize our knowledge of the efficacy of *Bs* MBI600 against these fungal and oomycete tomato pathogens under greenhouse or field conditions.

Measurements of tomato growth characteristics in plants treated with *Bs* MBI600 showed a strong ability to promote growth, leading to longer shoots and roots compared to untreated plants. A similar growth promotion ability of *Bs* MBI600 was previously observed in cucumber plants [25]. The growth promotion ability of *B. subtilis* or other *Bacillus* spp. has previously been reported in numerous studies on several hosts [19,20,30]. PGPR strains promote plant growth either via direct production of hormones such as auxin (indole-3-acetic acid, IAA), which are supplied to the host, or by regulating the expression of auxin-related host genes, leading to increased auxin production [31,32]. The modulation of a plant’s hormone production is a common strategy employed by PGPR strains to optimize their colonization on the roots of the host [33]. The selection of the three genes for the investigation of the effects of *Bs* MBI600 on auxin gene expression was based on previous findings suggesting that *PIN*, *AUX/LAX*, and *ARF* genes that play a predominant role in auxin fluxes in a wide variety of plant species, including tomato [34,35,36]. The increased expression rates of *SIPIN6* and *SILAX4* genes probably account for increased auxin production contributing to more rapid cell division, and subsequently higher growth rates for the *Bs* MBI600-treated plants. In addition to the induction of auxin production by PGPR, they can possibly promote plant growth through the enhancement of nutrient uptake by the colonized plant roots [37]. The annotation of the *Bs* MBI600 genome revealed the existence of a large number of genes involved in the enhancement of nutrient uptake and availability [25]. For instance, genes encoding nitrate and potassium transport or siderophore production were identified within the genome of this microorganism. However, further research is required to obtain full evidence on its contribution to tomato growth promotion through the enhancement of nutrient uptake. 

Several previous studies have shown that the biocontrol efficacy and plant growth promotion by PGPR microorganisms is affected by several factors, such as the growth system, the growth substrate and its physicochemical characteristics, and the colonization ability of the roots of the host [38,39,40]. In the current study, we took advantage of a previously transformed yellow fluorescent protein (yfp)-labeled strain with resistance to chloramphenicol, while population densities were measured on chloramphenicol-amended media [25]. The colonization ability of *Bs* MBI600 on tomato roots was tested on plants grown in four different substrates, which included both field conditions (natural vegetable soil and hydroponic cubes) and laboratory conditions (gnotobiotic system and commercial peat mixture). In our study, efforts were made to include substrates often used under realistic conditions of tomato cultivation, such as the natural vegetable soil or the hydroponic cubes. This is particularly important, as it has been shown in the past that differences in colonization ability under different conditions are major factors contributing to the restricted use of BCAs in the field [41]. Although soil substrates are the most common for tomato production under field or greenhouse conditions, the use of soilless substrates in hydroponic systems has started to become a standard practice for greenhouse-grown tomatoes throughout the world. However, even in hydroponic systems, zoosporic oomycete microorganisms such as *P. ultimum* or fungi that produce airborne microconidia such as *Forl* can still cause severe damage to tomato plants [5,7].

In all four substrates tested, *Bs* MBI600 was found to efficiently colonize tomato roots, although with variable rates. In all four substrates, the higher population densities were observed in the first sampling (five days post-application), while afterwards a reduction was observed. A similar pattern of a decline in population densities over time was observed in cucumber plants with *Bs* MBI600, which has also been reported in previous studies reporting the root colonization ability of *Bacillus* spp. on several hosts [25,38,41]. The host, with its exudates and root architecture, plays a dominant role in the colonization ability of the PGPR strains. In our study, although the pattern of changes in population densities was similar to those observed for cucumber roots grown on exactly the same substrates, on the tomato roots the *Bs* MBI600 population densities were lower than those observed on cucumber roots at the same time intervals [25]. Similar differences in population densities for the same PGPR on different hosts were previously reported for several *Bacillus* spp. [38,42,43]. The lower bacterial cell densities measured on the tomato roots were probably related to the specific characteristics of the tomato roots compared to those of cucumber plants. On cucumber plants, lateral roots are more abundant compared to those of tomato plants. This may explain the observed differences, since previous studies have shown that most *Bacillus* spp. form microcolonies on the surfaces of the outer epidermis cells of the primary root and at the junctions of primary and lateral roots [43,44].

Biocontrol mechanisms of plant pathogens include parasitism, competition, antibiosis, and induction of host resistance [45]. In our study, the antibiosis ability of *Bs* MBI600 was not tested directly. However, in vitro data on mycelial growth inhibition showed that *Bs* MBI600 was highly effective in reducing the mycelial growth of *Forl* and to a lesser extent of *P. ultimum*. These data suggest that *Bs* MBI600 may produce secondary metabolites that contribute to the suppression of the mycelial growth of *Forl.* Additionally, the genome analysis of *Bs* MBI600 has already revealed the presence of the gene clusters *srf* (A-B-C), *pps*A-*pps*E, *npr*, and *sbo*-*alb*, which encode surfactin, fengycin, bacilollycin, and subtolisin, respectively [25]. Further unpublished data from our group have confirmed fengycin and surfactin production by the BCA (Samaras, unpublished data). Several previous studies with a wide array of *Bacillus* spp. have shown that these PGPR strains are capable of exhibiting antagonistic and antibiotic activity by inhibiting the mycelial growth of several fungal species through the production of these secondary metabolites [46,47]. Further studies could unravel the full spectrum of secondary metabolites produced by *Bs* MBI600, which contribute to its antagonistic and antibiotic activity against plant pathogens.

A major aim of our study was to test whether *Bs* MBI600 applications may elicit defense responses on tomato plants in the absence and presence of a fungal pathogen and to gain insights into their molecular basis. In our experimental procedures, gene expression was measured on tomato roots, although it is well established from previous studies that plant defense signaling is less pronounced on root tissues compared to on shoots [48,49]. However, since in our study the targets of *Bs* MBI600 were soilborne pathogens and the roots of the plants are the initial infection sites, gene expression measurements were conducted on root tissues exposed to either *Bs* MBI600 or *Forl* and to both microorganisms. To determine the signaling pathways activated by *Bs* MBI600 applications in the presence or absence of a fungal pathogen, 5 defense-related genes that are considered as JA/ET signaling or SA signaling markers were selected. Among them, *LOXD*, *CHI3,* and *PAL* are considered as JA or ET signaling molecules, while *PR-1A* and *GLUA* are considered as SA signaling molecules [10,50,51,52].

Induction of defense-associated gene expression by *Bs* MBI600 was found to be weak during the early stages (24h pi) of root colonization, however at later stages (48 and 96 h pi) higher relative expression levels for genes such as *PR-1A*, *GLUA*, and *CHI3* were observed. On the other hand, for all five tested genes, significantly higher expression levels were observed in plants challenged only with *Forl.* This is consistent with findings of previous works suggesting that PGPR triggers only mild defense responses compared to those triggered by plant pathogens [15,53]. Interestingly, for some of the tested genes, their expression levels in plants challenged with both microorganisms was higher compared to in plants challenged only with *Forl*. This is in agreement with several previous studies suggesting that plant resistance to pathogens after exposure to *Bacillus* spp. is associated with priming effects [52,54]. Priming has been associated with surfactin and fengycin production using PGPR species. In addition to their direct roles in root colonization or antimicrobial activity, surfactin and fengycin have been shown to possibly mediate the communication with plants eliciting ISR [55,56,57]. As previously stated, unpublished data from our group have confirmed the production of fengycin and surfactin by *Bs* MBI600 (Samaras, unpublished data).

In our study, plants were exposed to the necrotrophic fungus *Forl*. Early reports suggested that JA/ET signaling was most effective in triggering defense responses against *Fusarium* spp; however, later on, several studies showed that SA signaling may also be effective against *Forl* or other *Fusarium* spp. [8,49,58]. Early responses of tomato when exposed to the pathogen or its combination with the BCA suggested an overexpression of genes associated with the JA/ET signaling pathway, such as *LOXD*, *CHI3*, and *PAL*. However, in more advanced infection stages on plants either inoculated with *Forl* or inoculated with *Forl* and treated with *Bs* MBI600, marker genes of the SA signaling pathway such as *PR-1A* and *GluA* were found to be upregulated, showing a 50–58- and 90–140-fold increases, respectively, in their expression levels. The observed activation of SA signaling on *Bs* MBI600-treated plants is in agreement with previous findings on tomato plants challenged with the same BCA and TSWV or PVY and in line with a previously defined crosstalk between SA and JA/ET pathways [27,51,59]. The synergistic activation of both JA/ET and SA signaling pathways mediating PGPR-imposed ISR has been previously shown in several other *Bacillus* strains on several plant hosts, including tomato plants [8,30,47,52].

In conclusion, in the current study, evidence was provided on the possible employment of *Bs* MBI600 as an eco-friendly approach to combat soilborne fungal and oomycete pathogens of tomato and to promote the growth of the plants, ensuring higher yield sustainability. *Bs* MBI600 was found to be able to modulate transcriptional activation of two auxin-related genes (*SiPin6* and *SiLax* 4). This activation, along with additional factors, may contribute to the growth promotion of tomato plants exposed to the BCA. Single applications of *Bs* MBI600 provided moderate control efficacy against the three major soilborne tomato pathogens tested. Transcriptional analysis of the expression patterns of SA and JA/ET signaling marker genes revealed a synergistic interaction of the two pathways, with increased expression on plants challenged with *Forl* and *Bs* MBI600. To our knowledge, this is the first study reporting the effects of this new BCA against soilborne tomato pathogens and examining the molecular mechanism behind its protective activity on tomato plants challenged with both the BCA and a fungal pathogen. These data suggest that *Bs* MBI600 possess great potential as a new alternative biocontrol agent that could be used in tomato crops cultivated both in soil or soilless substrates. However, its utility has to be confirmed with further experiments at the field or greenhouse level.

## 4. Materials and Methods

### 4.1. Plant Materials

Tomato cv. “Belladonna”, a cultivar susceptible to all major soilborne pathogens of tomato plants, was used in the study. Seeds were sown in 123-plug trays filled with a peat mix, covered by a vermiculite layer. Trays were watered regularly and kept under greenhouse conditions (20–25 °C with a 16/8 h photoperiod cycle and 60–70% RH). No pesticides or fertilizers were applied on the plants. Seedling plants at the 4th true leaf growth stage were used in the biocontrol experiments. 

### 4.2. Microorganisms 

The *Bs* MBI600 strain used in the study was isolated from a commercial formulation of the product (Serifel 9.9 WP, BASF Hellas), following a procedure described previously [25]. The isolated strain was maintained at −80 °C in tryptone soy broth (TSB, LabM, Hungary) supplemented with 40% glycerol. Before use, the bacterial culture was grown on tryptone soy agar (TSA) medium and incubated at 37 °C for 24 h.

*Fusarium oxysporum* f.sp. *radicis-lycopersici* (*Forl*), *Rhizoctonia solani*, and *Pythium ultimum* isolates used in the study belonged to the fungal isolates collection from the Lab of Plant Pathology, AUTH. All pathogens had been isolated from diseased tomato plants. The fungal isolates were grown and maintained on potato dextrose agar (PDA, LabM, Hungary) slants at 4 °C until use.

### 4.3. In Vitro Effects of Bs MBI600 against the Mycelial Growth of Soilborne Pathogens

The antagonistic activity of *Bs* MBI600 and its ability to arrest mycelial growth in vitro was determined against 3 major soilborne pathogens of tomato, namely *Forl*, *R. solani*, and *P. ultimum.* The in vitro effects of *Bs* MBI600 against mycelial growth was determined using the dual-culture technique [60]. Dual cultures on PDA medium consisted of the bacterial isolate and each of the 3 pathogen isolates inoculated on opposite sides of Petri dishes measuring 9 cm in diameter at approximately 10 mm distance from the margins of the Petri dish. The bacterial cells were streaked as a straight line onto the medium and the plates were inoculated with a 6-mm-diameter plug of mycelium taken from the colony margins of actively growing 7-day-old cultures of each pathogen. Plates were incubated for seven days at 25 °C and the antagonistic activity was evaluated by measuring the diameter of the pathogen colonies and the length of the inhibition zones (mm). Five replicate dishes were prepared per treatment and the experiment was repeated 3 times.

### 4.4. Plant Growth Promotion Assays

The effect of *Bs* MBI600 on the tomato growth was assessed by measuring the following growth and physiology parameters: shoot height, root length, shoot fresh weight, and root fresh weight. Tomato seeds were individually sown in plastic pots containing 80 cm^3^ of a 5:1 mixture of peat and perlite. Bacterial cultures were prepared in TSB-medium-containing flasks with shaking overnight at 37 °C. The suspension was then centrifuged at 4000× *g* for 5 min and the pellet was re-suspended in dd H_2_O until the OD (measured at 600 nm) of the culture reached values of 0.8. Then, 10 ml of the bacterial suspension was applied in each pot by soil drenching just after sowing. The application was repeated 20 days after sowing. In addition to *Bs* MBI600, the commercially available *Bacillus amyloliquefaciens* QST713 strain (Serenade ASO, 1.34SC, Bayer Crop Science, Monheim am Rhein, Germany), (hereafter *Ba* QST713) was included in the experimental design as a reference biological treatment. It was applied at the commercially recommended rate of 16 mL L^−1^ of formulated product. Control plants were drenched with distilled sterile water. Measurements of the growth parameters were conducted 15 days after the second application (35 days after seed sowing). During the experimental period, plants were maintained in greenhouse conditions at 20–25 °C with a 16/8 h photoperiod cycle and 60–70% RH. There were five replicates of 10 plants each in a completely randomized block design.

### 4.5. Root Colonization Assays in Various Growth Substrates

Colonization patterns of *Bs* MBI600 on tomato roots were tested in four different growth systems: sterile conditions (gnotobiotic system), commercial peat mixture, natural soil suitable for vegetable production (vegetable soil), and hydroponic cubes (Grodan, Roermond, The Netherlands). In all experimental procedures, a chloramphenicol-resistant and yellow fluorescence protein (yfp)-labelled *Bs* MBI600 strain was used [25]. The procedures followed on the four different growth systems were adjusted from methods applied previously on cucumber roots [25]. Samplings of roots were conducted at three time points, namely 5, 15, and 20 days after the application. Each root was placed into a tube with phosphate-buffered saline (PBS) and transferred in Elmasonic S30 to detach bacterial cells from the roots using ultrasonic waves at a frequency of 37 kHz. After appropriate dilution, the suspensions were plated onto Luria Broth plates amended with 5 ng mL^−1^ chloramphenicol. After 24 h of incubation at 37 °C, colonies were counted, the concentrations of which were calculated as cfu cm^−1^. To confirm that the isolated and counted bacterial colonies were those of the transformed *Bs* MBI600 strain, the inserted plasmid was detected in 10 randomly selected colonies per petri dish. Positive colonies were checked by fluorescence microscope and by colony PCR for the detection of the *yfp* gene inserted in pHCMC0_2_ plasmid during the transformation procedure using the yfpFw–yfpRv primer pair (Table 1).

### 4.6. In Planta Efficacy of Bs MBI600 for Controlling Soilborne Pathogens of Tomato

Tomato plants (cv. Belladonna) at the 4th true leaf stage were artificially inoculated with *Forl*, *R. solani*, and *P. ultimum*. For the production of *Forl* inoculum, mycelium was placed on PDA in 9 cm Petri dishes and incubated at 25 °C for seven days in darkness, following a procedure described previously [9]. Briefly, four mycelial plugs taken from 7-day-old cultures were transferred into 250 mL Czapek–Dox broth (CDB; Duchefa, Haarlem, The Netherlands) in 500 mL Erlenmeyer flasks and incubated for three days at 28 °C in a rotary shaker at 150 rpm. After filtration through four layers of cheesecloth, the concentration of the resulting spore suspension was estimated using a hemocytometer under light microscopy and adjusted to 5 × 10^5^ conidia ml^−1^. 

The inoculum of *P. ultimum* was prepared on 20% V-8 juice agar medium [61]. Plates were incubated at 25 °C in the dark for 10 days until sporangia production. Then, the cultures were blended for 30 s at high speed in a blender (Waring, New Hartford, NY, USA). Sporangia were counted with a hemocytometer and their concentration was adjusted at 5 × 10^3^ mL^−1^.

The *R. solani* inoculum for artificial inoculations was produced as described previously [62]. Briefly, a fungal culture was grown for seven days on PDA medium at 25 °C. Then, mycelia were dislodged by scraping the surface of the agar culture with a sterile glass rod. Suspensions were filtered through four layers of sterile cheesecloth and the fungal mycelium was transferred on flasks containing potato dextrose broth (PDB, LabM, Hungary). After incubation at 25 °C and 150 rpm for one week, the fungal mycelia were harvested by centrifugation at 11,000 rpm for 10 min and re-suspended in distilled water. The mycelium inoculum was concentrated at 5 × 10^7^ mycelial fragments mL^−1^.

For the artificial inoculation of the plants, each pot was drenched with 10 mL of the inoculum suspensions. Control plants were drenched with sterile distilled water. The application of *Bs* MBI600 was conducted once by drenching each pot with 15 mL of bacterial suspension (OD ~0.8) 24 h before the inoculation with the pathogens. In the experimental design, a standard chemical and a standard biological reference treatment were also included. *Ba* QST713 was the biological reference treatment applied at the commercially recommended dose of 16 mL L^−1^ formulated product, 24 h before the inoculation of the plants with the pathogens. 8-Hydroxyquinoline (Beltanol 37.5 SL, Agrology SA, Sindos, Greece) was the standard chemical treatment included in the experimental design against *P. ultimum* and *R. solani*, while hymexazol (Tachigaren 36SL, SUMITOMO Corp., Piraeus, Greece) was used as the reference chemical treatment against *Forl*. The application of fungicides was conducted 24 h before the artificial inoculation at the commercially recommended rates of 0.5 and 0.53 mL L^−1^ f.p. for 8-hydroxyquinoline and hymexazol, respectively. After artificial inoculations, plants were incubated for 15 days under greenhouse conditions (20–25 °C with a 16/8 h photoperiod cycle and 60–70% RH). For each pathogen, 3 replicates were performed, with 25 plants per treatment.

Disease assessments were conducted after the end of the incubation period. FCRR severity was measured using a 0–3 disease index scale as follows: 0 = uninfected plants; 1 = only secondary roots infected; 2 = main root infected; 3 = dead plants [63]. Rhizoctonia and Pythium damping-off severity were measured using a 0–1 disease index scale as follows: 0 = uninfected plants; 1 = dead plants.

### 4.7. Induction of Defense- and Auxin-Related Genes in Tomato Plants

To determine the transcript changes that took place in tomato roots after treatment with *Bs* MBI600, an experiment with tomato plants treated with the BCA and *Forl* was designed. Defense and auxin-related genes were chosen for expression analysis. For this purpose, three auxin-related genes (Silax4, SiArf4, and SiPin6) were selected, which represent the three major auxin-related gene families and are also localized in tomato roots and five genes (*PR-1a*, *GLUA*, *CHI3*, *LoxD*, and *Pal*) that were related to the most common defense mechanisms activated in plants after *Bacillus* treatments. 

Measurements of auxin- and defense-related genes expression was conducted on plants that received one *Bs* MBI600 application, while root samples for RNA extraction were collected 0, 24, 48, and 96 h post-application. In addition, measurements of defense-related genes expression were conducted on plants that received one *Bs* MBI600 application, which were artificially inoculated with *Forl* 24 h later. In these plants, root samples for RNA extraction were collected 0, 24, 48, and 96 h post-inoculation with the pathogen. The time points for the transcription analysis were selected according to the time required for *Bacillus* colonization and pathogen infection to increase the probability of detecting transcript changes [10,64].

Four biological replicates were used for each time point. Each sample comprised three plants. Samples were immediately placed on liquid nitrogen and stored at −80 °C until RNA extraction. Total RNA was extracted using the Trizol method according to manufacturer’s instructions (TRItidy G™, Germany). The qRT-PCR reactions were carried out on a StepOne Plus Real-Time PCR System (Applied Biosystems) using a SYBR-Green-based kit (Luna^®^ Universal One-Step RT qPCR Kit). Primers for this analysis are listed in Table S1.

### 4.8. Data Analysis

Data for the independent replications on plant growth parameters, disease incidence and severity, and bacterial cell enumeration in colonization experiments were combined after testing for homogeneity of variance using Levene’s test. The combined data were then subjected to one-way analysis of variance (ANOVA). Fisher’s LSD test was used for comparison of means. Gene expression data were normalized to the *cytochrome oxidase* (*cox*) gene expression and relative transcripts were calculated according to the 2^−ΔΔ*C*t^ method [65]. Statistical analysis was conducted using Tukey’s test. All statistical analyses were supported by SPSS 21.0 (SPSS, Chicago, IL, USA).

## Figures and Tables

**Table 1:** The effects of *Bacillus subtilis* MBI600 on the in vitro mycelial growth of the tomato pathogens *Fusarium oxysporum* f.sp. *radicis-lycopersici*, *Pythium ultimum*, and *Rhizoctonia solani* after seven days in dual culture.

Treatment	Pathogen								
	*Fusarium oxysporum* f.sp. *radicis* *lycopersici*			*Pythium ultimum*			*Rhizoctonia solani*		
	Colony Diameter (mm)	Relative Inhibition	Inhibition Zone ^a^	Colony Diameter (mm)	Relative Inhibition	Inhibition Zone	Colony Diameter (mm)	Relative Inhibition	Inhibition Zone
Control (Pathogen)	70b ^b^	0b	-	90b	0b	-	70b	0b	-
*Bs* MBI600 + Pathogen	25a	64.1a	++	65a	27.8a	-	65a	7.2a	+

^a^ Diameter (mm) of inhibition zone between pathogens and *Bs* MBI600 on PDA plates: - no inhibition; + inhibition zone of <10mm; ++ inhibition zone of >10 mm. ^b^ Mean values followed by different letters in the column indicate significant differences among treatments according to Fisher’s LSD test (*p* < 0.05).

**Table 2:** Counts (cfu cm^−1^) for chloramphenicol-resistant, YFP-tagged *Bacillus subtilis* MBI600 strain on tomato roots grown in 4 different growth substrates.

Growing System	Days after Application ^a^		
	5	15	20
Gnotobiotic system	2 × 10^5^ b ^b^ B ^c^	1.3 × 10^3^ bc B	3 × 10^2^ c A
Commercial Peat mixture	3.2 × 10^5^ b B	4 × 10^2^ c A	2.5 × 10^2^ c A
Vegetable soil	4 × 10^4^ b A	2 × 10^3^ bc B	1.7 × 10^2^ c A
Hydroponic cubes	3 × 10^5^ b B	2.4 × 10^3^ bc B	4 × 10^2^ c A

^a^ Initial application rate of *Bs* MBI600 was 2 × 10^10^ cfu mL^−1^. ^b^ Mean values followed by different lowercase letters in the rows indicate significant differences among days for each treatment according to Fisher’s LSD test (*p* < 0.05). ^c^ Mean values followed by different capital letters in the columns indicate significant differences among treatments according to Fisher’s LSD test (*p* < 0.05). Comparisons were made with the initial application rate of 2 × 10^10^ cfu ml^−1^.

## Data Availability

The data presented in this study are available in this article.

## Supplementary Materials

The following are available online at https://www.mdpi.com/article/10.3390/plants10061113/s1, Table S1: List of oligonucleotides used in the study.
