# Supplementary material for: Bacillus subtilis MBI600 Promotes Growth of Tomato Plants and Induces Systemic Resistance Contributing to the Control of Soilborne Pathogens
Source: Plants (Basel). 2021 May 31;10(6):1113. doi: 10.3390/plants10061113 (PMC8229581; doi:10.3390/plants10061113)
Supplement: Supplementary file 1 [file plants-10-01113-s001.zip › plants-1223936-supplementary.pdf]

Supplementary Table 1. List of oligonucleotides used in the study.

Gene name	Primer sequence (5′ – 3′)	Encoding protein	Signaling pathway	References
Silax4	TGCTGCAATTTAGACCTCTTTTT	Auxin	IAA/auxin	[1]
	GGATCCCAGTCGGTGTTC			
SiArf4	CATTATTGTTGGTGACTTTGTG	Auxin response factor	IAA/auxin	[2]
	GACCTTTGGAAACCTATTGG			
SiPin6	TCAATCAACCCTTTCACTTTCTT	Auxin	IAA/auxin	[1]
	GGGGGCCAAAGATTTTCTTA			
PR-1a	TCTTGTGAGGCCCAAAATTC	PR-1 (acidic type)	SA	[3]
	TAGTCTGGCCTCTCGGACA			
GLUA	GTCTCAACCGCGACATATT	PR-2 (β-1,3 glucanase	SA	[3]
	CACAAGGGCATCGAAAAG AT			
CHI3	TGCAGGAACATTCACTGGAG	PR-3 (Chitinase)	JA/ETH	[3]
	TAACGTTGTGGCATGATGGT			
LoxD	CCTGAAATCTATGGCCCTCA	Lipoxygenase	Oxylipin	[3]
	ATGGGCTTAAGTGTGCCAAC			
Pal	CGCTATGCT CTCCGAACATCTC	Phenyl ammonia lyase	JA/ETH	[4]
	ATTCACCGAGTTAATCTCCCTCTC			
CyOXID	TGGTAATTGGTCTGTTCC GATT	Cytochrome oxidase subunit I	Reference gene	[5]
	TGGAGGCAACAACCAGAATG			
yfp	ACATAAGGAGGAACTACTATGAGT	Yellow fluorescent protein	-	[6]
	GCGCTCACCCGGGTTATTTGTATAG			

1. Pattison, R.J.; Catalá, C. Evaluating auxin distribution in tomato (*Solanum lycopersicum*) through an analysis of the PIN and AUX/LAX gene families. *Plant J.* **2012**, *70*, 585–98.
2. Zouine, M.; Fu, Y.; Chateigner-Boutin, A.-L.; Mila, I.; Frasse, P.; Wang, H.; Audran, C.; Roustan, J.-P.; Bouzayen, M. Characterization of the tomato *ARF* gene family uncovers a multi-levels post-transcriptional regulation including alternative splicing. *PLoS ONE* **2014**, *9*, e84203.
3. Aimé, S.; Alabouvette, C.; Steinberg, C.; Olivain, C. The endophytic strain *Fusarium oxysporum* Fo47: A good candidate for priming the defense responses in tomato roots. *Mol. Plant Microbe Interact.* **2013**, *26*, 918–926.
4. Chandrasekaran, M.; Chun, S.C. Expression of PR-protein genes and induction of defense-related enzymes by Bacillus subtilis CBR05 in tomato (Solanum lycopersicum) plants challenged with Erwinia carotovora subsp. carotovora. *Biosci. Biotechnol. Biochem.* **2016**, *80*, 2277–2283.
5. Papayiannis, L.C.; Harkou, I.S.; Markou, Y.M.; Demetriou, C.N.; Katis, N.I. Rapid discrimination of Tomato chlorosis virus, Tomato infectious chlorosis virus and co-amplifcation of plant internal control using real-time RT-PCR. *J. Virol. Methods* **2011**, *176*, 53–5 9.
6. Caro-Astorga, J.; Álvarez-Mena, A.; Hierrezuelo, J.; Guadix, J.A.; Heredia-Ponce, Z.; Arboleda-Estudillo, Y.; González-Munoz, E.; de Vicente, A.; Romero, D. Two genomic regions encoding exopolysaccharide production systems have complementary functions in *B. cereus* multicellularity and host interaction. *Sci. Rep.* **2020**, *10*, 1000.
